# Visual Perturbation Suggests Increased Effort to Maintain Balance in Early Stages of Parkinson’s to be an Effect of Age Rather Than Disease

**DOI:** 10.3389/fnhum.2022.762380

**Published:** 2022-03-02

**Authors:** Justus Student, David Engel, Lars Timmermann, Frank Bremmer, Josefine Waldthaler

**Affiliations:** ^1^Department of Neurophysics, University of Marburg, Marburg, Germany; ^2^Department of Neurology, University Hospital of Marburg, Marburg, Germany; ^3^Center for Mind, Brain and Behavior (CMBB), University of Marburg and Justus-Liebig-University of Gießen, Marburg, Germany

**Keywords:** Parkinson’s disease, body sway, virtual reality, center of mass (CoM), center of pressure (CoP)

## Abstract

Postural instability marks a prevalent symptom of Parkinson’s disease (PD). It often manifests in increased body sway, which is commonly assessed by tracking the Center of Pressure (CoP). Yet, in terms of postural control, the body’s Center of Mass (CoM), and not CoP is what is regulated in a gravitational field. The aim of this study was to explore the effect of early- to mid-stage PD on these measures of postural control in response to unpredictable visual perturbations. We investigated three cohorts: (i) 18 patients with early to mid-stage PD [Hoehn & Yahr stage (1–3); 1.94 ± 0.70]; (ii) a group of 15 age-matched controls (ECT); and (iii) a group of 12 young healthy adults (YCT). Participants stood on a force plate to track their CoP, while the movement of their entire body was recorded with a video-based motion tracking system to monitor their CoM. A moving room paradigm was applied through a head-mounted virtual reality headset. The stimulus consisted of a virtual tunnel that stretched in the anterior-posterior direction which either remained static or moved back and forth in an unpredictable fashion.We found differences in mean sway amplitude (MSA) and mean velocities of CoP and CoM between the groups under both conditions, with higher MSA of CoP and CoM for PD and higher mean velocities of both variables for PD and ECT when compared with YCT. Visual perturbation increased mean CoP velocity in all groups but did not have effects on mean CoM velocity or MSA. While being significantly lower for the young adults, the net effect of visual perturbation on mean CoP velocity was similar between patients with PD and age-matched controls. There was no effect of the visual perturbation on mean CoM velocity for any of the groups.Our simultaneous assessment of CoP and CoM revealed that postural control is reflected differently in CoM and CoP. As the motion of CoM remained mostly unaffected, all groups successfully counteracted the perturbation and maintained their balance. Higher CoP velocity for PD and ECT revealed increased corrective motion needed to achieve this, which however was similar in both groups. Thus, our results suggest increased effort, expressed in CoP velocity, to be an effect of age rather than disease in earlier stages of PD.

## Introduction

Due to the ongoing demographic transition and subsequent over-aging of modern societies in many industrialized countries, neurodegenerative diseases are gaining in prevalence, with Parkinson’s disease (PD) being the second most common after Alzheimer’s disease (Lange and Erbguth, 2017; Tysnes and Storstein, 2017). In addition to the cardinal symptoms of rigidity, hypokinesia and resting tremor, as the disease progresses, most patients also develop postural instability (Jankovic, 2008). The onset of postural instability, as determined by clinical tests, can vary widely between individuals and ranges from months to decades after initial diagnosis. Postural instability comprises the inability to maintain equilibrium under dynamic and static conditions such as preparation of movements, perturbations, and quiet stance (Appeadu and Gupta, 2021) and leads to an increased risk of falls. Reportedly, postural instability is associated with a reduction in quality of life and even diminished life expectancy of those affected (Koller et al., 1989; Fasano et al., 2017; Bäckström et al., 2018). However, since only some patients exhibit a progressive form with severe instability, it is crucial to identify those who are prone to falls. A common indicator to predict the individual risk of future falls is whether the patient had experienced more than two falls in the preceding year (Pickering et al., 2007). This implies, however, that patients already had a history of falls and thus hampers the usefulness as a predictor to take preventive measures. Thus, identifying reliable biomechanical indicators of postural instability is crucial to monitor the development of this symptom and allow early intervention.

Maintaining equilibrium, i.e., a stable upright body position in space, involves complex, intertwined processing of various sensory inputs. Out of these inputs, visual information is claimed to be one of the most important contributors (Berthoz et al., 1975; Bronstein, 1986; Laurens et al., 2010). Sense of balance can easily be manipulated by external visual stimuli, which in turn has the potential to give insight into the underlying neural processing (van Asten et al., 1988; Alghadir et al., 2019). Regarding such visual manipulations, previous research has shown that unpredictable perturbations of the visual scene generate a strong dynamic destabilization of a quiet upright stance (Winter et al., 1990; Guerraz et al., 2001; Musolino et al., 2006; Barela et al., 2009). In particular, patients with PD show an increased reliance on the visual system to maintain an upright stance as compared to healthy individuals (Bronstein et al., 1990; Weil et al., 2016). Moreover, the stimulus used in this study was found to successfully produce different responses in patients with PD and age-matched controls (Engel et al., 2021b). Based on these previous results, a visual disturbance was added in this study with the aim to increase potential differences between PD and ECT.

A sensitive measurable indicator of this destabilization constitutes an increased sway of the body (Horak and Mancini, 2013; Pantall et al., 2018). The body’s Center of Mass (CoM) is claimed to be the main variable controlled by the central nervous system to maintain equilibrium (Horak and Macpherson, 1996; Peterka, 2002). However, considering the heterogenic mass distribution of the human body, assessment of CoM requires tracking and subsequent mathematical processing of several body segments. Therefore, tracking of the Center of Pressure (CoP), which reflects the total of forces enacted on the ground, is widely used in body sway research, as its motion can directly be assessed through simple force platforms (Vsetecková and Drey, 2013). Existing models to describe the relationship between CoP and CoM during human bipedal upright stance include that of an inverted pendulum (Winter et al., 1997; Gage et al., 2004; Laurens et al., 2010). In this context, CoM typically is considered to be the controlled variable, while CoP is the controlling variable. Accordingly, CoP controls CoM and keeps its position above the base of support (Winter et al., 1996; Takeda et al., 2017; Morasso, 2020). Displacements of CoM which exceed this area lead to falls if the current velocity is directed away from the center (Horak et al., 1989; Hof et al., 2005; Roman-Liu, 2018).

The velocity of these body sway parameters has proven to be a suitable measure to compare body sway between particular groups with balance impairments and healthy controls (Takeda et al., 2017), while the sole displacement of their trajectories is suggested to be less suitable to identify potential differences (Masani et al., 2014). For instance, Palmieri and colleagues showed that an increase in CoP velocity signifies increased postural instability while slower CoP velocity indicates more effective balance control in an upright stance (Palmieri et al., 2002). Moreover, Nantel and colleagues showed that higher CoP velocities seem to be an indicator of the severity of postural instability in PD. Their study revealed that age-matched controls are able to maintain their posture more effectively, signalized by a lower CoP velocity (Nantel et al., 2012). Thus, CoP velocity might serve as a potential biomarker for deterioration of postural stability (Maurer et al., 2003; da Conceição et al., 2019) and might predict fallers in PD (Beretta et al., 2018). However, there is evidence that increased CoP velocities also occur in the healthy elderly (Roman-Liu, 2018). Since most people affected by PD are of advanced age (Rijk et al., 1995), it is unclear whether this deterioration of postural control is due to the disease alone.

Due to the hitherto cumbersome assessment of CoM, the majority of existing studies only investigated the motion of CoP. CoM is claimed to be the main controlled variable by the central nervous system, as it represents the interaction of the body with the gravitational field. Even though recent technologies allow for easier tracking of the whole body, research on CoM motion, especially in response to visual perturbations, remains sparse. Moreover, in this way, tracking of CoM provides insight into actual 3-D motion of the body, as opposed to COP, which only describes a 2-D projection on the ground Thus, investigation of CoM motion bears the potential to investigate additional alterations that might be related to PD as well as age.

Recently, affordable, and user-friendly equipment, which was originally designed for the gaming industry, has been validated for research purposes. This includes the Microsoft Kinect v2 (Microsoft, Redmond, WA, USA), which allows for video-based full-body motion tracking, as well as the Nintendo Wii Balance Board (Nintendo, Kyoto, Japan) to replace a research-grade force platform (Chang et al., 2013; Dehbandi et al., 2017; Clark et al., 2018). In addition, a convenient means to perform visual manipulations that has recently been established is the use of commercially available virtual reality headsets to apply a moving room paradigm (Engel et al., 2020, 2021a). Accordingly, a combination of these experimental tools and approaches enables a straightforward simultaneous assessment of CoP and CoM under visual perturbations, through which empirical conclusions can be drawn about the relationship between both parameters.

Thus, the aim of this study was to simultaneously assess CoP and CoM dynamics to investigate differences in mean sway amplitude (MSA) and mean velocity of both parameters between patients with early-to-mid stage PD (PD), a control group (ECT) of age-matched healthy adults and a third group of young (YCT) healthy adults. To assess potential differences which occur before the onset of recurrent falls due to postural instability, we purposely recruited patients in early to mid-stages of the disease. This study may hence provide a proof-of-concept to demonstrate the feasibility of postural assessment using a low-cost set-up in patients with PD. By this means, we aim to lay a foundation for the long-term goal of our research which is to establish nuanced and sensitive measures to detect PD patients at risk of falls before the onset of clinically apparent postural instability.

Postural behavior was evaluated under two conditions: (i) during quiet standing, while participants’ visual field remained stable; and (ii) while they underwent unpredictable perturbations of their visual surroundings in the anterior-posterior (AP) direction. By testing three different groups, our goal was to differentiate between potential disease-specific and age-related alterations in body sway during quiet stance as well as under visual perturbation, which might be expressed differently in CoP and CoM. We hypothesized: (1) that patients with PD show the largest MSA and highest velocity of both parameters under both conditions, followed by the elderly healthy adults, while both parameters will be smallest for the young group. Moreover, we expected to find (2) that MSA and mean velocity of both parameters increase under visual perturbation in all groups. Lastly, we hypothesized (3) that these increases will again be strongest for the group of patients and weakest for the group of young healthy adults.

## Materials and Methods

### Participants

Eighteen patients with PD [age: range: (42–76); mean ± standard deviation: 58.10 ± 8.66] diagnosed based on the Movement Disorder Society diagnostic criteria (Postuma et al., 2015) in early to moderate disease stages {Hoehn and Yahr: [(1–3); 1.94 ± 0.70; Hoehn and Yahr, 1967] with a mean disease duration of 4.8 years [(0–15); 4.79 ± 4.71]} participated in the study. A prerequisite for the inclusion of patients was that they were able to walk without any assistance and did not report more than one fall in the previous year. Only one patient reported a previous fall event. Also, none of the patients had experienced *Freezing of Gait* before or during the experiment. All of the patients were assessed “on” their regular dose of dopaminergic medication [Levodopa Equivalent Daily Dose (LEDD): (105–1980); 651.63 ± 529.97]. The two control groups consisted of 15 age-matched healthy individuals [age: (49–70); 59.80 ± 6.45] as well as 12 young healthy participants [age: (22–28); 23.92 ± 1.50].

Exclusion criteria were any neurological disorder other than PD (e.g., neuropathies, epilepsy, multiple sclerosis, schizophrenia, severe, depression, dementia, etc.) or orthopedic (e.g., at the hip, spine, knee, etc.) disorders which could affect their balance and upright stance as well as cognitive impairment based on the Montreal Cognitive Assessment as a screening tool for general cognitive abilities with a cut-off score of 24 points (Ciesielska et al., 2016). All subjects had normal or corrected to normal visual acuity.

All participants gave written informed consent prior to the experiment, including with regard to the storage and processing of their data. Experimental procedures involving healthy individuals were approved by the Ethics Committee of the Psychology Department, University of Marburg. Research including patients with PD was approved by the Ethics Committee of the Faculty of Medicine, University of Marburg (Case 77/19). All research was conducted in accordance with the Declaration of Helsinki.

### Technical Setup

**Figure 1:**
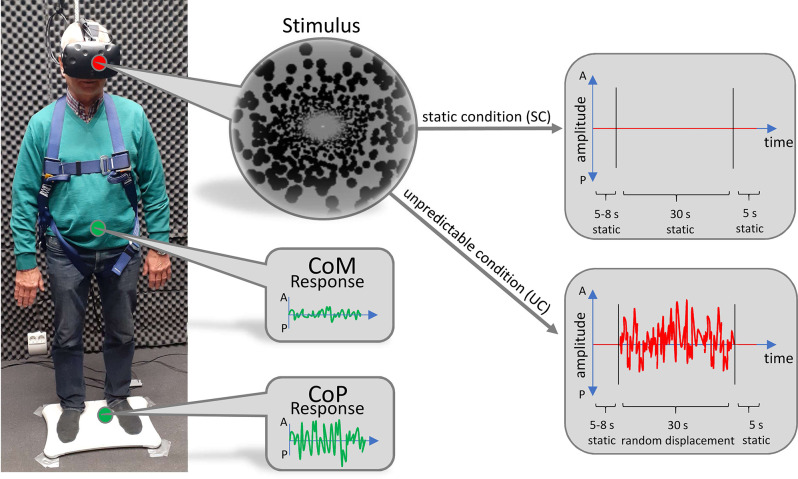
Technical setup. Subjects stood on a Wii Balance Board to track their CoP. Their body movement was tracked by a video-based motion tracking system positioned in front of them (not displayed). Visual stimuli were presented through a head-mounted virtual reality headset. Stimuli consisted of a virtual tunnel in anterior-posterior (AP)-direction, which either remained static (upper right panel) or moved back and forth unpredictably (lower right panel).

Participants stood on a Wii Balance board (Nintendo, Kyoto, Japan) to track their CoP (Figure 1). Wearing no shoes, they were instructed to position their feet about shoulder-width apart, about parallel on the ground. During trials, their arms were to hang at their sides without effort. They were instructed to remain their gaze straight ahead. To perform tracking of their body motion, we used a Kinect v2 video-based motion tracking system (Microsoft, Redmond, WA, USA) which recorded the 3-D positions of 25 different “body joints” as determined by an internal algorithm. The camera was located 210 cm in front of the participants and fixed at a height of 140 cm. Visual stimuli were presented through a head-mounted virtual reality headset (HTC Vive, HTC, New Taipei City, 206 Taiwan). The frame rate was 90 Hz. The field of view extended over 110° in the vertical as well as horizontal directions.

In the virtual world, participants stood inside a tunnel that was world-fixed and stretched in the AP direction. The visual stimulus was custom made, based on the open-source Python pyopenvr framework^1^. The demarcation of the tunnel was made up of black spheres, whose position was randomly generated for every trial. The length of the tunnel was set to 50 m and the radius was adjusted to the eye level of every subject so that their gaze was directed at the radial center of the tunnel, 25 m in front of them. Just before the end of the tunnel, at a distance of 24 m, we implemented a fixation dot at the radial center.

To prevent participants from falling, they were secured by a harness which was attached to the ceiling. We ensured that the harness guaranteed subjects’ safety but was not providing lift during trials. The setup has successfully been established and described in more detail in previous work (Engel et al., 2021a, b).

### Experimental Paradigm

Each subject performed 10 trials of the static condition (SC) where the tunnel remained motionless and 20 trials of the unpredictable condition (UC), during which the tunnel was moving back and forth in an unpredictable fashion. In this way, every subject performed a total of 30 trials. The order of trials was pseudorandomly shuffled. The unpredictable motion of the tunnel was made up of a series of random tunnel displacements along the AP axis which were based on a flattened random frequency spectrum (random white noise). This was to ensure that all frequencies were equally present in the sequence, which improved comparability between trials and avoided biases towards specific frequencies that might have interfered with the body sway of individual subjects. The maximum step size was set to 80 cm. At the beginning of each trial, the tunnel remained motionless and—in the UC—started moving after a randomized onset time between 5 and 8 s. The motion lasted for 30 s, after which the tunnel remained static for another 5 s to provide the subjects with relaxation time. In this way, the duration of each trial was 40–43 s.

As soon as the subjects indicated by a verbal command that they were ready, the experimenter started the next trial. To signal its beginning, the fixation dot changed its color from red to white. During the 30 s motion phase (or static phase), the fixation dot occasionally shifted its color to black in a transient fashion. This event occurred randomly between zero and 10 times over the course of each trial. Participants’ task was to pay attention to these shifts and keep track of their numbers to ensure that they kept fixating. After the end of each trial, subjects had to report the number of changes they noticed. If the reported number was off by more than two, the respective trial was discarded. After each trial, subjects had time to relax and adjust their posture as long as they needed. Across all trials, every subject had a minimum of two larger breaks to prevent fatigue. During these breaks, participants were able to leave the setup and sit down or walk around. The entire experiment took about 2 h to complete.

### Data Analysis

A custom-made Python script was used for recording and storing raw data. Further analyses were conducted in MATLAB (The MathWorks, Inc., Natick, USA). For all following analyses, we extracted the time-courses of CoP and the 3-D body segments which corresponded to the AP-directions. Time courses of the CoP, which more precisely reflect the displacement, and the 3-D body segments from individual trials were aligned to the respective stimulus onset.

To determine the position of CoM, we calculated the 3-D center positions of 16 body segments and summed them with individual weighting factors taken from Winter (2009, p. 86). Before determining our final variables, in each trial, we subtracted a first-order polynomial from the raw data of CoP and CoM using the detrend function in MATLAB. This was to eliminate potential continuous motion artifacts which might have occurred during trials (Cruz et al., 2021). To determine the MSA, we calculated the standard deviation of the detrended time courses. Afterward, we computed the mean velocity by taking the first derivative of the detrended time courses and calculated the average of the absolute values of the derivation across time. In order to investigate additional temporal dynamics of the obtained MSA and mean velocities, we also distributed the detrended signals into small bins of 0.5 s, of which we calculated MSA and mean velocity individually.

Lastly, to gain insight into the net effect of the unpredictable condition on the motion of CoP and CoM we subtracted MSA values obtained during the static condition from the MSA values obtained during visual perturbation for each parameter, respectively. In the same way, we calculated the net effect of the visual perturbation on the mean velocities of both parameters.

### Statistics

We used non-parametric testing on ranked data due to our unequal group size, the relatively small number of participants, and subsequent non-comparable variance. Kruskal-Wallis tests with follow-up pairwise comparisons using Dunn’s test with Bonferroni correction were used to evaluate group differences (PD, ECT, YCT). Differences between groups according to *post hoc* pairwise comparisons are reported as effect sizes based on correlation coefficients (r) using z-standardized test statistics. To test the influence of the unpredictable movement of the tunnel on MSA and the mean velocities of CoP and CoM within each group, we performed a Wilcoxon signed-rank test. We considered 95% confidence intervals (*p* < 0.05) to reject the null hypothesis. Statistical analyses were performed in SPSS (IBM, Armonk, NY, USA).

## Results

**Figure 2:**
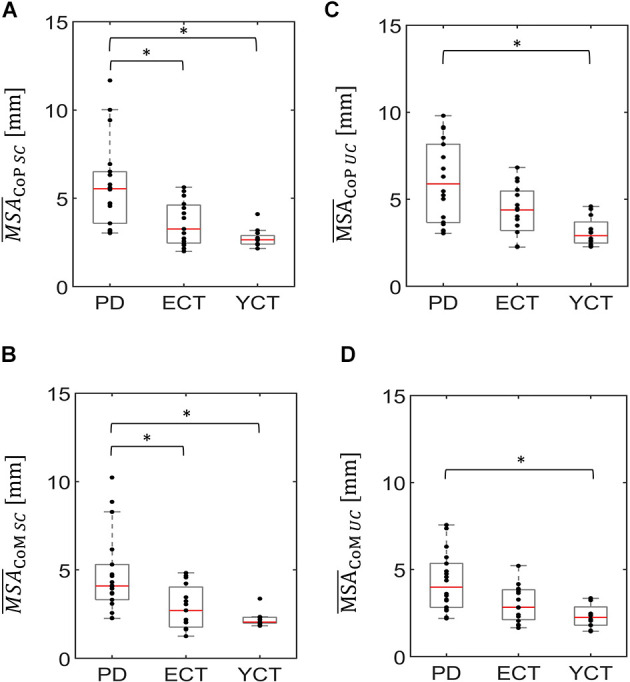
Box plots representing the mean sway amplitude of CoP (panel **A**) and CoM (panel **B**) of all groups under the static condition (SC) and the mean sway amplitude of CoP (panel **C**) and CoM (panel **D**) of all groups under the unpredictable condition (UC), when the tunnel moved unpredictably in the AP direction. In each plot, red lines indicate the respective group medians. Gray boxes indicate the 25th (lower edge) and 75th (upper edge) percentiles of each group, respectively. Black dots indicate data of single subjects within each group. The asterisks indicate that there is a significant difference between the groups. CoP, Center of Pressure; CoM, Center of Mass. ^*^p < 0.5.

**Figure 3:**
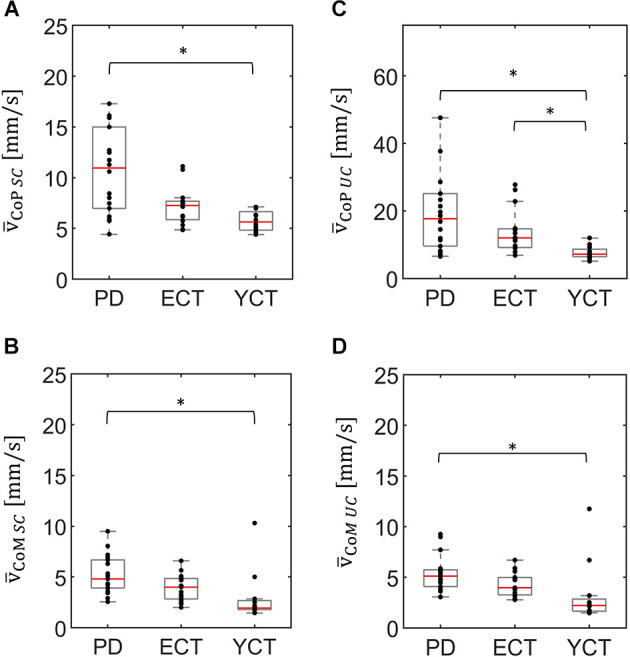
Box plots representing the mean velocity of CoP (panel **A**) and CoM (panel **B**) of all groups under the static condition (SC) and the mean velocity of CoP (panel **C**) and CoM (panel **D**) of all groups under the unpredictable condition (UC), when the tunnel moved unpredictably in the AP direction. In each plot, red lines indicate the respective group medians. Gray boxes indicate the 25th (lower edge) and 75th (upper edge) percentiles of each group, respectively. Black dots indicate data of single subjects within each group. Note the different scaling of panel **(C)** as compared to panels **(A,B,D)**. ^*^p < 0.5.

In a first step, we analyzed baseline data, i.e., MSA and velocity of CoP and CoM while the tunnel remained static (SC). There was a significant effect of group on the mean CoP sway amplitude (*H*_(2)_ = 20.21, *p* < 0.001) and on mean CoP velocity (*H*_(2)_ = 14.91, *p* = 0.001). As displayed in Figure 2A, there was a visible trend across groups, with patients with PD showing the highest mean CoP sway amplitude, followed by the ECT group, which in turn exhibited a slightly higher mean CoP sway amplitude than the YCT group. In *post hoc* pairwise comparisons, there were significant differences between PD and ECT (*p* = 0.009, *r* = 0.51) and between PD and YCT (*p* < 0.001, *r* = 0.79). However, there was no difference between ECT and YCT (*p* = 0.41, *r* = 0.29). A similar trend became apparent in mean CoP velocities, displayed in Figure 3A. However, there were neither significant differences between PD and ECT (*p* = 0.255, *r* = 0.35) nor between ECT and YCT (*p* = 0.376, *r* = 0.36). Only the higher mean CoP velocity of PD deviated significantly from the mean CoP velocity of the YCT group (*p* = 0.001, *r* = 0.70).

Regarding mean CoM sway amplitude (Figure 2B), there was also a significant effect of group (*H*_(2)_ = 17.157 *p* < 0.001). Here, a similar trend emerged as in the CoP data. *Post hoc* comparisons revealed significant differences between PD and ECT (*p* = 0.013 *r* = 0.50) and between PD and YCT (*p* < 0.001 *r* = 0.72). There was no statistically significant difference between the YCT and ECT (*p* = 0.68 *r* = 0.23). Further, the effect of group was also significant for mean CoM velocities (*H*_(2)_ = 14.288, *p* = 0.001), accompanied by a similar trend as for CoP (Figure 3). Here, there was also no significant difference between PD and ECT groups (*p* = 0.351, *r* = 0.27), while CoM of YCT moved significantly slower in comparison to PD (*p* < 0.001, *r* = 0.69). It also moved considerably slower than CoM of the elderly healthy adults, albeit not reaching statistical significance (*p* = 0.079 *r* = 0.43).

In a second step, we compared responses to the unpredictable visual movement of the tunnel between the groups in terms of MSA (CoP: *H*_(2)_ = 14.42, *p* = 0.001; CoM: *H*_(2)_ = 13.63, *p* = 0.001) and mean velocities (CoP: *H*_(2)_ = 14.76, *p* = 0.001; CoM: *H*_(2)_ = 13.25, *p* = 0.001). The results are displayed in panels C and D of Figures 2, 3. Here, a similar trend across groups like in the static condition (SC) became apparent. The PD group showed the highest mean CoP sway amplitude, followed by ECT. Mean CoP sway amplitude of the YCT was the lowest. In contrast to the SC, we were only able to detect statistically significant differences between PD and YCT (*p* < 0.001, *r* = 0.69), but neither between PD and ECT (*p* = 0.21, *r* = 0.32) nor between ECT and YCT (*p* = 0.13, *r* = 0.39). Furthermore, like in the SC, we did not observe statistical difference in mean CoP velocity between PD and ECT (*p* > 0.999, *r* = 0.17). However, there were significant differences between a higher mean CoP velocity of PD as compared to YCT (*p* = 0.001, *r* = 0.69) as well as between higher values of ECT as compared to YCT (*p* = 0.034, *r* = 0.53). Investigation of mean CoM sway amplitudes during the unpredictable condition showed the same trend. There was a significant difference between PD and YCT (*p* = 0.001, *r* = 0.67). However, neither PD and ECT (*p* = 0.138, *r* = 0.347) nor ECT and YCT (*p* = 0.256, *r* = 0.33) exhibited significant differences in their CoM MSA. The same applied to the mean CoM velocities. Here, there was also only a significant difference between the PD and YCT groups (*p* = 0.001, *r* = 0.66).

In addition to the differences between groups in both the SC and the UC, we also investigated the effect that the visual influence had on each group. For the mean CoP sway amplitude under visual perturbation, we were able to find an increase for both the ECT (*p* = 0.004, *r* = 0.75) and the YCT (*p* = 0.023, *r* = 0.66) groups when compared with the SC. On the other hand, we could not find any difference between the different conditions within the PD group (*p* = 0.616, *r* = 0.12). Mean CoM sway amplitudes were not influenced by the visual perturbation.

Unpredictable motion of the tunnel significantly increased mean CoP velocity in all groups when compared with the static condition (PD: *p* = 0.001, *r* = 0.81; ECT: *p* = 0.001, *r* = 0.88; YCT: *p* = 0.002, *r* = 0.88). This was also observed in the mean velocity of CoM, albeit to a much smaller extent. Here, statistical tests only yielded significant effects for ECT (*p* = 0.001, *r* = 0.84) and YCT (*p* = 0.041, *r* = 0.59).

**Figure 4:**
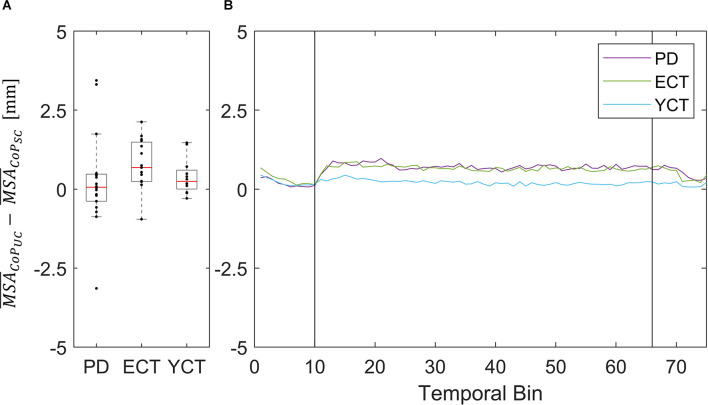
**(A)** Box plots showing the net effect of the unpredictable tunnel motion on mean CoP sway amplitude as compared to the static condition for each group. Red lines indicate the respective group medians. Gray boxes indicate the 25th (lower edge) and 75th (upper edge) percentiles of each group, respectively. Black dots indicate data of single subjects within each group. **(B)** Net-effect “time courses” of resulting binned mean CoP sway amplitude across time. Black vertical lines indicate stimulus onset (left) and offset (right).

**Figure 5:**
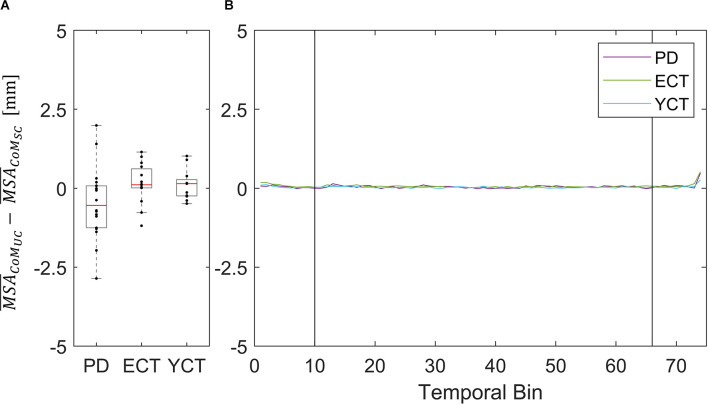
**(A)** Box plots showing the net effect of the unpredictable tunnel motion on mean CoM sway amplitude as compared to the static condition for each group. Red lines indicate the respective group medians. Gray boxes indicate the 25th (lower edge) and 75th (upper edge) percentiles of each group, respectively. Black dots indicate data of single subjects within each group. **(B)** Net-effect “time courses”of resulting binned mean CoM sway amplitude across time. Black vertical lines indicate stimulus onset (left) and offset (right).

**Figure 6:**
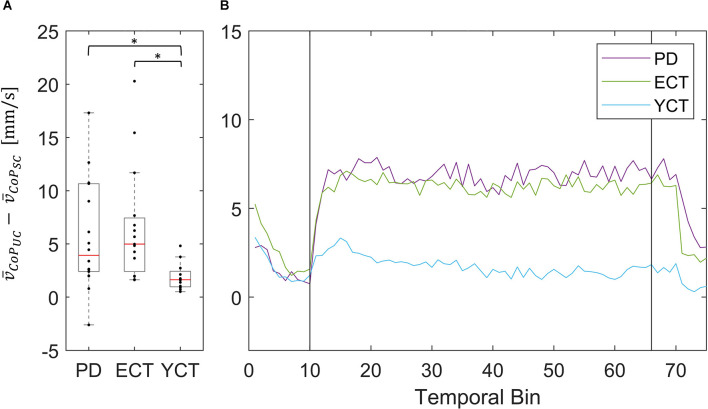
**(A)** Box plots showing the net effect of the unpredictable tunnel motion on mean CoP velocities as compared to the static condition for each group. Red lines indicate the respective group medians. Gray boxes indicate the 25th (lower edge) and 75th (upper edge) percentiles of each group, respectively. Black dots indicate data of single subjects within each group. The asterisks indicate that there is a significant difference between the groups. **(B)** Net-effect “time courses” of resulting binned mean CoP velocities across time. Black vertical lines indicate stimulus onset (left) and offset (right).

**Figure 7:**
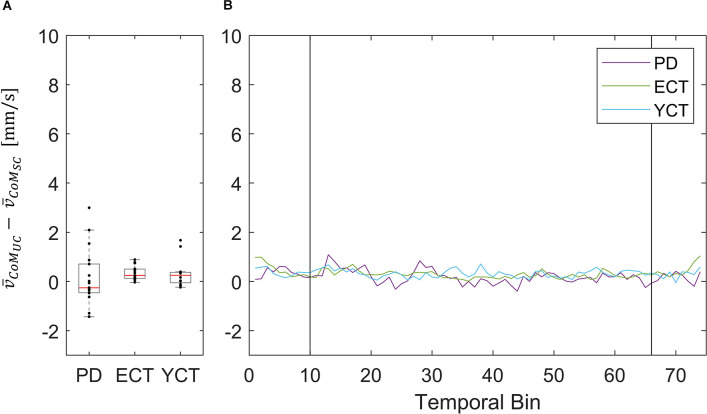
**(A)** Box plots showing the net effect of the unpredictable tunnel motion on mean CoM velocities as compared to the static condition for each group. Red lines indicate the respective group medians. Gray boxes indicate the 25th (lower edge) and 75th (upper edge) percentiles of each group, respectively. Black dots indicate data of single subjects within each group. **(B)** Net-effect “time courses” of resulting binned mean CoM velocities across time. Black vertical lines indicate stimulus onset (left) and offset (right).

As the motion of the tunnel led to an increase in mean velocity in each respective group and of MSA of CoP in ECT and YCT, we also investigated the net effect of visual perturbation (UC) on MSA and mean velocity of both parameters. To this end, for each group, we subtracted the average MSA during the static condition from the average MSA during perturbation. The same was done for the mean velocities. Even though in the case of CoM the increase in both parameters was rather small, for comparison, we also calculated the net effect of visual perturbation on CoM MSA and velocity. The results for the MSA data are displayed in panel A of Figure 4 (CoP) and Figure 5 (CoM), respectively, and the result for the mean velocities are shown in panel A of Figure 6 (CoP) and Figure 7 (CoM). Despite the apparent trend, we could not find an effect of the group on MSA, neither for CoP nor for the CoM data (CoP: *H*_(2)_ = 5.41, *p* = 0.067 and CoM: *H*_(2)_ = 6.06, *p* = 0.048). Even when Kruskal-Wallis testing of CoM data yielded *p* < 0.05, this could not be confirmed in the subsequent pairwise comparisons with corrected p-values.

Nevertheless, the net difference revealed that visual perturbation led to a strong increase of mean CoP velocity in both PD and ECT, while it had only a slight effect on YCT. This was backed by statistical testing, which yielded unpredictable visual perturbation to have a significantly different net effect on mean CoP velocity between PD and YCT (*p* = 0.013, *r* = 0.52) as well as between ECT and YCT (*p* = 0.006, *r* = 0.59). There was no significant difference between PD and ECT (*p* > 0.999, *r* = 0.06). To gain additional insight into the temporal dynamics of this effect, we sorted the mean CoP and CoM velocities of each group in temporal bins, as described in our methods. The resulting binned time series for mean CoP velocity can be seen in Figure 6B. Shortly after stimulus onset, mean CoP velocity increased drastically in both elderly groups (PD and ECT), but only minimally for the young. Remarkably, the rise in CoP velocity occurred with the same latency for PD and ECT, i.e., roughly 300 ms. The latency in the YCT was lower at around 100 ms. During the stimulus, each group maintained a considerably stable mean CoP velocity. When the motion of the tunnel stopped, again with similar latency (2.5 s), the mean CoP velocity of PD and ECT dropped to the prestimulus level.

This did not apply to the difference in mean velocities of CoM between the two conditions, as there was barely a visible net effect in any of the groups. This was also reflected in statistical testing between groups which revealed no significant effects. The net effects on mean CoM and their respective binned distribution over time are displayed in Figure 4.

## Discussion

In our study, we investigated potential disease- and age-specific changes in postural control during quiet stance in a static environment (static condition, SC) as well as in response to random visual perturbations (unpredictable condition, UC) by simultaneously assessing mean sway amplitude and mean velocity of CoP and CoM. For this purpose, we recruited three different cohorts: a group of patients with PD (PD), a group of age-matched healthy adults (ECT), and a group of young healthy adults (YCT).

Considering mean sway amplitude and mean velocity of both CoP and CoM, we hypothesized the highest amount of body sway for the group of patients with PD in both visual conditions, SC and UC, respectively. This was confirmed for MSA in both parameters which corroborates previous research (e.g., Bronstein et al., 1990; Cruz et al., 2018), whereas the observable trend in mean velocity data was not reaching statistical significance.

In terms of the effect of visual perturbation on body sway, we could only find increased mean CoP sway amplitudes for ECT and YCT but not for PD. There was no effect of the visual perturbation on mean CoM sway amplitudes in any of the groups. Based on our velocity data, we were able to partially confirm our second hypothesis, since all groups exhibited a higher mean velocity of their CoP when exposed to random displacements of the tunnel. However, this was again only true for CoP in the PD and ECT groups and did not hold for CoM in any of the groups. Thirdly, we expected that the net increase would again be strongest for the group of patients. Even though we found differences between groups, this hypothesis was not supported by our findings.

In general, CoP MSA was higher than CoM MSA in all groups, irrespective of the condition. These findings reflect the fact that displacement of CoP needs to exceed that of CoM in a given direction in order to properly counteract its movement (Winter, 2009; Zemková et al., 2016; Takeda et al., 2017). The same was true for the mean velocity of CoP, which was higher than the mean velocity of CoM in each group. This is in line with previous research, which revealed a higher dynamic range of CoP when compared to CoM (Winter, 1995; Winter et al., 1996; Horak et al., 2005).

In addition, across groups, there were the same trends regarding MSA and mean velocities of CoP and CoM under both conditions (Figures 3, 4). Even though our data revealed a slight increase in CoP MSA for the ECT and YCT groups, MSA, in general, remained almost unaffected by the visual stimulation for both parameters. However, our data on mean CoP velocity revealed that it almost doubled for PD and ECT when exposed to the visual perturbation, and still increased slightly for the YCT group. Furthermore, the mean CoM velocity was barely affected by the visual perturbation in any of the groups. When compared to displacement (MSA) as a measure of postural sway, our findings suggest the mean velocity of CoP to better reflect postural destabilization due to the movement of the tunnel. Accordingly, as our stimulus and thus the perturbation quickly changed directions, it seems logical that CoP had to move more quickly, reflected in a higher velocity, to keep CoM stable. This is remarkable and supports the idea that CoP and CoM show different behavior in response to a destabilizing environment (Carpenter et al., 2010; Takeda et al., 2017).

The increased motion of CoP but unaffected motion of CoM could be explained by the two different functions these variables are claimed to represent in the context of balance control. As described in our introduction, CoM is suggested to be the variable controlled by the CNS to maintain equilibrium (Winter et al., 1990; Horak et al., 2005; Zemková et al., 2016). It, therefore, reflects the outcome of internal processes as a response to the sensory input and a slow overall velocity indicates general stability. As visual perturbation barely affected the motion of CoM, this can be interpreted that all the groups successfully maintained their balance. The motion of CoP, on the other hand, reflects the forces enacted on the ground to push the body (CoM) back towards its equilibrium position once it gets deflected. Hence, the increased motion of CoP, expressed in our study as mean velocity, might represent a certain kind of effort that was required to perform its task.

Although we could detect higher CoP MSA for PD when compared to ECT, to our surprise, we did not find significantly higher CoP or CoM velocities between patients with PD and age-matched controls. However, there was high variation within the group of patients, which might have masked possible effects. In order to find potential indicators for postural instability at the early and mid-stages of PD, we purposely selected a high proportion of patients who only had mild symptoms. Clinical postural instability, defined as H&Y 3, had only previously been diagnosed in a few patients included in our study (Table 1). Thus, our results might suggest that balance control as expressed in CoP and CoM velocity is not impaired in these early stages, which was in line with the clinical assessment of our patients. This result of our study indicates that these parameters may be unsuitable to identify early disease-related changes of postural instability.

**Table 1:** Demographic information regarding age (in years), Montreal Cognitive Assessment (MoCA) score, Hoehn & Yahr stage (H & Y stage), levodopa equivalent dose (LEDD), and duration of the disease since initial diagnosis of each individual subject in the PD group.

Patient ID	Age (years)	MoCA	H & Y—Stage	LEDD (mg)	Disease duration (months)
1	55	27	2	900	79
2	51	30	1	105	29
3	70	26	2	282	9
4	52	29	2	250	5
5	51	29	1	242	15
6	62	26	3	821	86
7	53	29	2	935	176
8	70	28	3	210	40
9	50	29	3	774	62
10	55	28	1	150	21
11	53	28	2	786	43
12	68	26	3	1,880	188
13	53	29	2	1,980	133
14	76	24	2	465	32
15	65	28	1	515	61
16	57	29	2	780	32
17	42	29	1	121	2
18	63	28	2	532	33

On the other hand, mean CoP sway amplitude and velocity were lower for the group of young healthy adults. A similar effect could be seen in the CoM data. With CoM as an outcome of balance control mechanisms, both older groups exhibited greater instability compared to the younger adults. This indicates increased CoP and CoM velocity, both reflecting poorer balance control, to be an effect of age rather than disease (Masani et al., 2014; Roman-Liu, 2018). Since we used visual perturbation in our paradigm, this can possibly be explained by increased reliance on vision not only by the patients with PD, but by the elderly in general (Hay et al., 1996), but further experiments are required to settle this question.

In addition to our investigation of CoP and CoM displacements (MSA) and velocities between groups under both conditions separately, we also investigated the net effect of the visual perturbation on both parameters inside each group (Figures 4–7). There was no net effect on MSA, neither of CoP (Figure 4A) nor of CoM (Figure 5A). Regarding the net effect on CoP velocity (Figure 6), the differences between the groups as compared to the separate conditions became more pronounced. Here, the difference of data between both elderly groups and the young participants reached statistical significance, while there was again no significant difference between data from patients with PD and their age-matched peers. The net effect revealed a strongly visible gap between the elderly and the young (Figure 6), again speaking in favor of an age effect of increased CoP motion in response to the visual perturbation. By sorting the time-course data into bins and calculating MSA and mean velocity for each bin, we were able to probe the temporal dynamics of both parameters during stimulus presentation. Even though visual inspection of the mean of the binned data suggests an effect on CoP MSA in PD and ECT (Figure 4B), there was no statistical correlation. Accordingly, none of the groups exhibited an effect of the visual stimulation on the MSA of both parameters.

With regard to mean velocity, temporal dynamics did not only confirm the difference in behavior between both elderly groups and the young adults, but now also revealed that CoP velocity increased at a very short latency with respect to stimulus onset for all groups (Figure 4B). Moreover, it also decreased again with a similar delay for all groups. Hence, there seemed to be no measurable difference in response time between the groups, neither due to age nor due to disease. This is contrary to the evidence on prolonged latencies in response to motor tasks in PD (De Nunzio et al., 2007), but at the same time in line with other studies, which also did not detect differences in neural response latency (Rinalduzzi et al., 2015). A direct comparison of the net effects of the visual disturbance between MSA and mean COP velocity shows that only mean CoP velocity increases significantly due to the destabilizing effect of the unpredictably moving stimulus.

To our surprise, there was barely a noticeable net effect on CoM mean velocity, also considering temporal dynamics (Figure 7). None of the groups exhibited any destabilization in their CoM, as its velocity did not increase under visual perturbation. This means, as we stated before, that by increasing the motion of their CoP all groups were able to maintain their CoM at equilibrium and thus successfully counteracted the visual perturbation. In this regard, there was again neither an effect of age nor of disease on postural stability. The majority of patients (83%) in the PD group were in Hoehn & Yahr stages 1 or 2, i.e., exhibiting no postural instability in clinical examination. Only four of the patients were in H&Y stage 3. Hence, our results suggest that measuring CoM may accurately reflect the clinical state. In light of the differential results regarding CoM and CoP, the joint measurement of both variables adds significant value to exploring the equilibrium behavior. In addition, the inexpensive and easy-to-use alternative for measuring both CoP and CoM used in this study significantly simplified data collection compared with previous approaches for measuring CoM. Therefore, it would be intriguing to perform longitudinal studies with larger cohorts to also look into possible effects of disease progression on our measured parameters and to define a narrower time frame as to when balance deterioration starts to occur in PD.

The main limitations of our study include the relatively small and heterogenous groups. In a *post hoc* sample size calculation, the strong effect size regarding the influence of the mean CoP velocity by the stimulus compared to static condition translated into the minimum required sample sizes (PD: *n* = 15, ECT: *n* = 13, YCT: *n* = 13) that were satisfied by the number of participants in each group. Still, there is a risk that the study may have been underpowered for detecting meaningful differences in other measures between older healthy controls and participants with PD considering the trends in the data that did not reach statistical significance (for example, in mean CoP velocity). Furthermore, there was a rather large variety of disease duration, motor disease severity, and dosage of dopaminergic medication in the PD group. On the other hand, considering the large variability within PD itself regarding disease progression, this sample represents a typical cohort of PD patients in the early and mid-disease stages. Only three patients were diagnosed with postural instability, while the majority were in the early stages of the disease. However, despite postural impairment being claimed to only become present in advanced stages of the disease (Jankovic, 2008), we hoped to find impairments in balance control which were not yet manifested as apparent clinical symptoms. Moreover, all patients were treated with different doses of dopaminergic medication depending on their symptoms. Although the impact of dopaminergic medication on postural stability in general as well as on CoP velocity remains an area of debate as previous studies found conflicting results (Rocchi et al., 2002; Maurer et al., 2003; Nantel et al., 2012), we cannot exclude effects of medication on our results. Furthermore, no additional clinical scores (e.g., UPDRS-III) were recorded to more precisely capture motor symptom severity and balance impairment in relation to our findings.

The exact position and distance between the feet on the Wii Balance Board has not been specified which might have influenced CoP measurements between trials (Chiari et al., 2002; Palmisano et al., 2020). Lastly, although previous studies did not support a significant effect of a dual-task design on CoP displacement in PD (Fernandes et al., 2015), maintaining balance under additional cognitive load caused by the counting task might have affected our findings.

## Conclusion

In our study on visual perturbation of balance in PD and two healthy control groups, we found effects of visual perturbation on CoP dynamics, but only weak effects on CoM dynamics, which could be explained by the different natures of both parameters. These effects were much stronger for patients with PD and age-matched controls than they were for young healthy adults, which supports previous findings on the deterioration of balance with age. Against our expectations, we did not identify subclinical alterations of visuomotor balance control in patients with early- to mid-stage PD. Instead, we found similar behavior in both elderly groups when exposed to our unpredictable visual perturbations. Nonetheless, in light of their limitations, our findings suggest that the mean velocity of CoP may provide a useful quantitative measure to objectify clinical findings of balance control while experiencing a non-stationary visual scenery.

## Data Availability Statement

The raw data supporting the conclusions of this article will be made available by the authors, without undue reservation.

## Ethics Statement

The studies involving human participants were reviewed and approved by Ethics Committee of the Psychology Department, University of Marburg and Ethics Committee of the Faculty of Medicine, University of Marburg (Case 77/19). The patients/participants provided their written informed consent to participate in this study. Written informed consent was obtained from the individual(s) for the publication of any potentially identifiable images or data included in this article.

## Author Contributions

JS: conceptualization, formal analysis, investigation, data curation, writing—original draft, and visualization. DE: conceptualization, methodology, software, validation, formal analysis, writing—review and editing, visualization. LT: validation, writing—review and editing. FB: conceptualization, validation, resources, writing—review and editing, supervision, project administration, and funding acquisition. JW: conceptualization, validation, resources, writing—review and editing, supervision, and project administration. All authors contributed to the article and approved the submitted version.

## Conflict of Interest

The authors declare that the research was conducted in the absence of any commercial or financial relationships that could be construed as a potential conflict of interest.

## Publisher’s Note

All claims expressed in this article are solely those of the authors and do not necessarily represent those of their affiliated organizations, or those of the publisher, the editors and the reviewers. Any product that may be evaluated in this article, or claim that may be made by its manufacturer, is not guaranteed or endorsed by the publisher.

## Footnotes

^1^ https://github.com/cmbruns/pyopenvr
